# A probabilistic atlas of the human ventral tegmental area (VTA) based on 7 Tesla MRI data

**DOI:** 10.1007/s00429-021-02231-w

**Published:** 2021-02-12

**Authors:** Anne C. Trutti, Laura Fontanesi, Martijn J. Mulder, Pierre-Louis Bazin, Bernhard Hommel, Birte U. Forstmann

**Affiliations:** 1 Integrative Model-Based Cognitive Neuroscience Research Unit, University of Amsterdam, Nieuwe Achtergracht 129, 1018 WS Amsterdam, The Netherlands; 2 Cognitive Psychology Unit and Leiden Institute for Brain and Cognition, Leiden University, Leiden, The Netherlands; 3 Psychology Department, Center for Economic Psychology and Decision Neuroscience, University of Basel, Basel, Switzerland; 4 Utrecht University, Experimental Psychology, Utrecht, The Netherlands; 5 Departments of Neurophysics and Neurology, Max Planck Institute for Human Cognitive and Brain Sciences, Leipzig, Germany

**Keywords:** Probabilistic atlas, 7 T MRI, VTA, Midbrain, Subcortex

## Abstract

Functional magnetic resonance imaging (fMRI) BOLD signal is commonly localized by using neuroanatomical atlases, which can also serve for region of interest analyses. Yet, the available MRI atlases have serious limitations when it comes to imaging subcortical structures: only 7% of the 455 subcortical nuclei are captured by current atlases. This highlights the general difficulty in mapping smaller nuclei deep in the brain, which can be addressed using ultra-high field 7 Tesla (T) MRI. The ventral tegmental area (VTA) is a subcortical structure that plays a pivotal role in reward processing, learning and memory. Despite the significant interest in this nucleus in cognitive neuroscience, there are currently no available, anatomically precise VTA atlases derived from 7 T MRI data that cover the full region of the VTA. Here, we first provide a protocol for multimodal VTA imaging and delineation. We then provide a data description of a probabilistic VTA atlas based on in vivo 7 T MRI data.

## Introduction

Neuroanatomical atlases can be used to localize functional magnetic resonance imaging (fMRI) BOLD signal in univariate (voxel-wise) analyses, or to extract BOLD signal in the region of interest analyses (Poldrack 2006). Currently, only 7% of the 455 subcortical nuclei are included in the available magnetic resonance imaging (MRI) atlases. This highlights the general difficulty in mapping smaller subcortical structures (Alkemade et al. 2013; Forstmann et al. 2017). One prominent subcortical nucleus is the ventral tegmental area (VTA). The VTA is located in the midbrain and contains dopaminergic neurons, which are crucial in reward-based learning and motor functions (Schultz 1998, 2015). Although some digital VTA atlases are available for neuroimaging purposes, there is a lack in availability of anatomically precise atlases derived from high-resolution 7 Tesla (T) MRI data that emphasize taking into account individual anatomical variability and precision.

Due to anatomical variability in the subcortex (Keuken et al. 2014), creating probabilistic atlases that capture this variability is crucial and particularly important for the VTA. The VTA is relatively small, with a size of circa 140 mm^3^, has a complex shape (Halliday and Törk 1986), and lacks a clear anatomical border with the surrounding nuclei. For all these reasons, delineating the VTA on individual MRI scans is very challenging. Despite these challenges, some efforts have been made to provide digital VTA atlases (Murty et al. 2014; Pauli et al. 2018). Eapen et al. (2011) and Ballard et al. (2011) set remarkable groundwork for segmenting the VTA on MRI data and Murty et al. (2014) provided the first publicly available probabilistic atlas of the VTA. Recently, Pauli et al. (2018) released another probabilistic atlas of the VTA by making use of the large publicly available data the field has gathered since. Accordingly, the atlas by Pauli et al. (2018) was aimed to emphasize anatomical variability, but interestingly the authors also applied a different VTA terminology compared to Murty et al. (2014).

The atlas introduced in this paper adds to the collection of probabilistic VTA atlases by providing another atlas that emphasizes anatomical precision next to anatomical variability which was the focus of previous atlases. Importantly, we acknowledge previous efforts; however, there are several challenges in the construction of VTA atlases that can be facilitated today due to developments in the field of ultra-high-field MRI. For example, given the relatively small size and location of the VTA, a scan protocol with a submillimeter voxel-size resolution is crucial (Ewert et al. 2018). Also, a 7T scan protocol helps to acquire improved signal-to-noise ratios (SNR) and contrast-to-noise ratios (CNR) compared to lower field strength (van der Zwaag et al. 2015). Furthermore, the optimization of the MR sequences by means of tailoring them to subcortical structures such as the VTA is required for individual anatomical precision (Trutti et al. 2019).

Finally, the interindividual anatomical variability of the VTA necessitates a large number of manual delineations by different raters separately for both hemispheres, respectively, to account for anatomical variability as well as the reliability of the segmentation.

Of course, all efforts to provide probabilistic VTA atlases are limited by some factors and therefore tailored to different approaches. In the atlas of Pauli et al. (2018) anatomical variability was taken into account before manual segmentation was carried out by means of computing eight, unilateral group templates on which three raters manually segmented the VTA. This resulted in eight, unilateral VTA segmentations for each rater despite the abundance of individual data, as the data originated from the Human Connectome Project (HCP). Yet, manual segmentations are very time consuming and the authors provided many subcortical nuclei in the published atlas. Thus, their efforts were not limited to segmenting the VTA. Further, since they worked with HCP data, they were also restricted to the available scans. To get images that allow segmentation of subcortical nuclei, the construction of group templates was required.

In contrast, the atlas from Murty et al. (2014) is based on manual segmentation of the VTA on a large number of participants. Nonetheless, as the field rapidly develops the data quality is not state-of-the-art anymore. The resolution (1 mm isotropic) and CNR of the used T1-weighted MRI sequence render the incorporation of small anatomical differences needed for the segmentation of the VTA very challenging. These issues can be addressed again today with new scan protocols and higher field strengths.

Besides the use of digital atlases, researchers who aim to provide anatomical precision in their neuroimaging efforts are limited to the use of topological, histological atlases such as the Mai, Majtanik, and Paxino’s atlas of the human brain (Mai et al. 2016). However, VTA representations in digital and topological atlases differ substantially not only due to interindividual variability in anatomy but also due to differences in VTA terminology (see Trutti et al. 2019 for a comprehensive review). Another factor that might influence the lack of agreement in terminology, and consequently the variability in VTA representations in the aforementioned atlases, is the structure’s cytoarchitectonic and neurochemical heterogeneity. This heterogeneity is not restricted to the VTA’s neural populations but is also apparent in its lack of a clear anatomical border, which makes it difficult to define boundaries in structural and histological studies and explains the poverty in available probabilistic VTA atlases.

In this study, we first present an optimized 7T MRI imaging protocol to delineate the VTA based on a well-established VTA terminology (Trutti et al. 2019). According to Halliday and Törk (1986) as well as Mai et al. (2016), the VTA covers a region that includes the parabrachial pigmented nucleus (PBP), paranigral nucleus (PN), interfascicular nucleus (IF), the caudal linear nucleus (CLi), and the rostral linear nucleus (RLi). Hence, the segmentation protocol was built so that the VTA masks could contain the different VTA component nuclei rather than to identify the individual component nuclei themselves, which is hardly possible even at 7T. We then demonstrate the capabilities of this optimized protocol to define a probabilistic VTA atlas based on 7T in vivo MRI data from 27 healthy participants.

## Data, materials and methods

### Participants

The probabilistic atlas is based on twenty-seven healthy, young, right-handed participants (19 females) with a mean age of 24.5 (SD 4.8). All participants had normal or corrected-to-normal vision, and none of them had a history of neurological, major medical, or psychiatric disorders. The study was approved by the local ethics committee at the University of Amsterdam, the Netherlands. All participants gave their written informed consent prior to scanning and received monetary compensation for participating. This study was performed in line with the principles of the Declaration of Helsinki.

### Scan parameters

The structural data were acquired using a Philips Achieva 7T MRI scanner equipped with a Nova Medical 32-channel head array coil. T1-weighted and T2*-weighted images, along with other images, were simultaneously obtained using a multi-echo magnetization-prepared rapid gradient echo (MP2RAGE-ME) sequence (Caan et al. 2018; Metere et al. 2017) with a total acquisition time of 19.53 min (TI,1 = 670 ms, TI,2 = 3675.4 ms, TR,1 = 6.2 ms, TR,2 = 31 ms, TE,1 = 3 ms, TE,2 = [3, 11.5, 19, 28.5 ms], TR, MP2RAGE-ME = 6778 ms, flip angle1: 4^◦^, flip angle2: 4^◦^, bandwidth: 404.9 MHz, acceleration factor SENSE: 2, FOV = 205 × 205 × 164 mm^3^, acquired voxel size: 0.7 × 0.7 × 0.7 mm^3^, acquisition matrix: 292 × 290, reconstructed voxel size: 0.64 × 0.64 × 0.70 mm^3^, turbo factor: 150 (resulting in 176 shots).

### Image processing

**Fig. 1:**
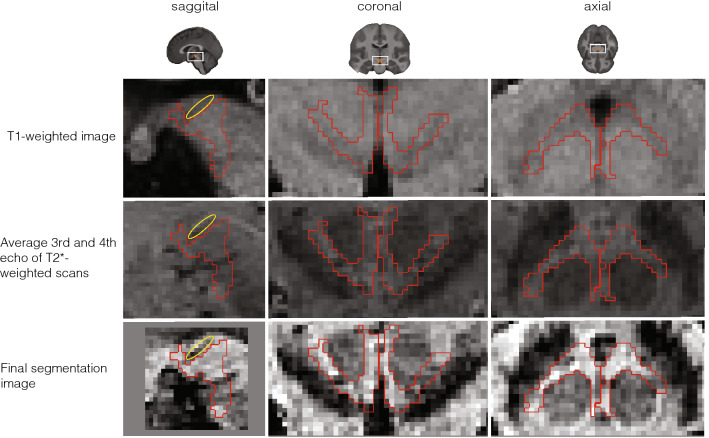
Detail of the midbrain area of one individual participant for each of the different scans collected for VTA delineation. The figure displays VTA (red) for one participant in the sagittal (left column), coronal (middle column), and axial (right column) plane. The first row shows the T1-weighted image of the first inversion of the MP2RAGEME sequence. The second row shows the image based on the average between the third and fourth echo of the T2*-weighted scans. And the third row depicts the final image used for VTA delineation, which was obtained by normalizing the image in the first and second row within the midbrain area (see 2.3). A blood vessel passing through the midbrain is highlighted in yellow, which was avoided during segmentation

Because the T1-weighted and T2*-weighted structural images were acquired simultaneously using the MP2RAGE-ME sequence (Caan et al. 2018; Metere et al. 2017), co-registration was not necessary. All obtained scans were individually checked for sufficient contrast in the VTA region to identify sequences that allow VTA delineation. To first locate and then manually delineate the VTA even though it lacks a clear border, we used its surrounding structures as landmarks and virtual borders. These included the fourth ventricle, the red nucleus (RN), and the substantia nigra (SN). This procedure is in line with histology approaches in which VTA delineations are also based on neighboring structures (Mai et al. 2016), as well as previous MRI-based delineations (Murty et al. 2014; Ballard et al. 2011). To increase contrast in our regions-of-interest (ROIs), we performed a few preprocessing steps prior to the manual delineation, as the visibility of the VTA and its neighboring structures varied across the three different scan contrasts (Fig. 1). While the fourth ventricle was best visible in the T1-weighted image, images from the second inversion of the T2*-weighted scans provided best visibility of both the SN and the RN. Since different landmark structures were visible in different images, we set up a procedure to include all of the landmark structures in the same image. Precisely, three images were chosen, across which the visibility of each structure varied: the T_1_-weighted image and two inversions of the T^∗^_2_-weighted scan, namely the 3rd and 4th echo. Additionally, for each subject, a midbrain area (with volume 1.6 × 1.6 × 3.08 cm^3^) was defined, so that it included the VTA as well its neighboring landmarks (i.e., the RN, the SN, and the 4th ventricle). The preprocessing steps thus included (1) averaging the 3rd and 4th echo of the T_2_^*^-weighted scan to increase the signal-to-noise ratio (SNR); (2) intensity-normalization over the voxels of the pre-defined midbrain area of the previously computed average T_2_^*^-weighted scan and of the T_1_-weighted image; (3) summing the normalized, T1-, and T2*-weighted midbrain sections. The image resulting from step 3 (‘midbrain contrast') was ultimately used to delineate VTA at the subject level.

### Registration to standard stereotactic MNI152 2009b space

All linear registration steps were done using MIPAV 5.4.4. (http://mipav.cit.nih.gov/) with the optimized automated linear registration algorithm. Whole-brain images were skull-stripped using the standard FSL BET tool (Smith 2002). The registration to atlas space was done by means of non-linearly aligning the individual whole-brain images to a group average template of the AHEAD database (Alkemade et al. 2020) which itself was co-registered to the MNI152 2009b template with the ANTs algorithm (Avants et al. 2008), using conservative deformation parameters as recommended for subcortex (Ewert et al. 2018). The individual VTA masks and midbrain contrasts were non-linearly transformed into MNI152 2009b space using the computed transformations. All registration steps were visually checked for misalignments by comparing several landmarks: fourth ventricle, pons, corpus callosum, and lateral ventricles. Because the AHEAD template is based on the same MP2RAGE-ME sequence that provides additional subcortical contrast compared to the MNI152 2009b template, the automated co-registration of the subcortical regions was satisfactory.

### Delineation protocol

**Fig. 2:**
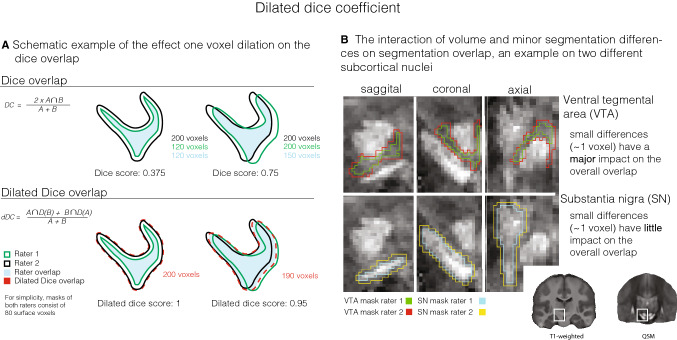
Dice and dilated Dice coefficient. The Dice coefficient is a spatial overlap index that by means of manual segmentation in MRI serves as a reproducibility validation metric (Zou et al. 2004). In the case of subcortical nuclei, a reduced border contrast often complicates manual segmentation substantially, especially for complex-shaped and relatively small structures such as the VTA. **a** If many voxels represent border/surface voxel, as in the VTA, a difference of one voxel at the border has a huge weight on the overall agreement. The dilated Dice score is a more flexible measure for inter-rater agreement and is therefore a more meaningful reproducibility metric for complex shapes as compared to the classical Dice score. In the equation, the letter A represents the mask of rater 1 and B the mask of rater 2. **b** The more voxels represent surface voxels in relation to the number of voxels of that structure in total (the surface/volume ratio) the more important exact overlap between raters becomes, as the classical Dice score penalizes each non-overlapping voxel. The example is shown on QSM images of a single subject with the VTA represented in the upper panel and the SN in the bottom panel. For orientation purposes a T1-weighted as well as a QSM whole-brain image is shown in the bottom right corner

Manual delineation was carried out by two independent researchers (LF, ACT) using the FSL 3.2.0 viewer (http://fsl.fmrib.ox.ac.uk/fsl/fslwiki/). Only voxels rated by both raters as belonging to the VTA were included to compute the VTA probabilistic atlas. Anatomical landmarks and the segmentation protocol were based on the work of Halliday and Törk (1986), van Domburg and ten Donkelaar (1991), Ballard et al. (2011) and Murty et al. (2014). The manual delineation was done as follows: in an initial step, the individual T1-weighted image of the MP2RAGE-ME sequence was loaded for orientation purposes, as the final image for delineation contained only the midbrain section (‘pre-defined midbrain area’ see 2.3). In a second step, the final, previously computed midbrain image was loaded and overlaid on the whole-brain T1-weighted image, and served as a basis for delineation. In a third step, the contrast values in the viewer for the delineation image were set to increase the visibility of the structural borders. The contrast values were determined on an individual subject basis and were independently set by each rater, but starting with a minimum of − 2 and maximum of + 2. For most participants, these initial contrast settings were found optimal for manual delineation. The contrast values were kept constant between hemispheres. The coronal view was picked to start delineating the structure. Once the delineation of the structure started, all three views were used to segment the structure. The order in which the right or left hemisphere was segmented was randomized per participant. First step of the manual segmentation involved identifying the starting point, the peak curvature of the white matter tract that connects the mamillary bodies with the midbrain (in sagittal view). In a second step, the voxels considered to be part of the VTA were segmented. The main body of the VTA lies between the relatively easily identified landmarks RN (dorsally), SN (ventro-laterally), the aqueduct (medially) and the fourth ventricle (ventro-medially). The subthalamic nucleus, if present, represents a much harder to identify dorsolateral landmark. Finally, the mask volume was computed and the interrater reliability was assessed by means of calculating different similarity metrics: the Dice coefficient, Hausdorff distance, and dilated Dice coefficient. The Dice coefficient represents the degree of spatial overlap between raters and is computed based on the size of the conjunct mask of the masks by rater 1 and rater 2, respectively, which only includes voxels included by both raters’ segmentations (see Fig. 2a for the equation to calculate the Dice coefficient). The Hausdorff distance is a metric that indicates how far shapes are from being isometric, while taking into account both shape and orientation. The dilated Dice coefficient, a more suited measure of reliability for small structures with complex shapes is similar to the classical Dice coefficient except that it calculates the degree of overlap based on dilating each mask by one voxel. Accordingly, it does not penalize a single-voxel offset to the same degree as the classical Dice score, which is an important factor for small, complex-shaped structures with a large ratio of surface voxels, like blood vessels or the VTA (see Fig. 2; Bazin et al. 2016). Interrater agreement is reflected in high (dilated) Dice values (1 = full agreement, 0 = no agreement), and a small Hausdorff distance. All statistics were derived using the Nighres toolbox (Huntenburg et al. 2018; https: //nighres.readthedocs.io-/en/latest/index.html) and its ‘segmentation_statistics’ function.

### Computation of probability maps and atlasing of the VTA

Manual delineations were carried out in individual spaces. To compute a probabilistic atlas, individual VTA masks were later registered to the group space as reported in “Registration to standard stereotactic MNI152 2009b space”. Consequently, the statistical atlas was generated by averaging each individual segmented VTA mask in standard space.

### Differences to other probability atlases of the VTA

To compare the currently available probabilistic atlases of the VTA with the atlas introduced in this manuscript, we moved to the standard space. The atlases of Murty et al. (2014) and Pauli et al. (2018) were registered to the MNI152 2009b template using the ‘embedded_antsreg’ (Avants 2008; Gorgolewski et al. 2011) and ‘apply_coordinate_mappings’ functions and consecutively, overlap statistics were computed using again the ‘segmentation_statistics’ function from the Nighres toolbox (Huntenberg et al. 2018).

## Results

The individual, manually delineated VTA masks had an average size of 137.35 mm^3^ (SD = 38.27) and 138.80 mm^3^ (SD = 39.33), for the left and for the right hemisphere, respectively. An ANOVA on the effects of rater and hemisphere on the volume of the individual delineations revealed a statistically significant main effect of rater, *F*(1, 104) = 36.57, *p* < 0.001, but no main effect of hemisphere, *F*(1, 104) = 0.11, *p* = 0.739, nor an interaction between rater and hemisphere, *F*(1, 104) = 0.68, *p* = 0.412. Levene’s test did indicate heteroscedasticity in the volume measure, but a Shapiro–Wilk test on the ANOVA residuals found no indication that normality is violated.

**Fig. 3:**
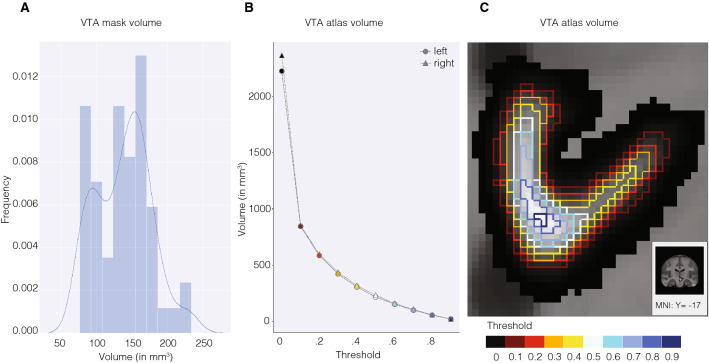
VTA mask and atlas volume. **a** Distribution of the volume of individual VTA conjunction masks. **b** VTA atlas volume across different thresholds. The size of the VTA atlas decreases when discarding the voxels that have lower probabilities of belonging to the VTA across participants. Markers are colored according to the thresholds in **c**. **c** Effect of thresholding on the VTA atlas volume, shown on the right VTA atlas. Voxels inside the differently colored outlines fall within the atlas mask when the thresholds corresponding to those colors are applied. For example, most of the darker voxels suggesting a lower probability, and only those, would be excluded with a threshold of 0.2 as indicated by the bright red outline. In contrast, picking a high threshold (blue colored lines) would mostly include voxels in the ventromedial VTA region

**Fig. 4:**
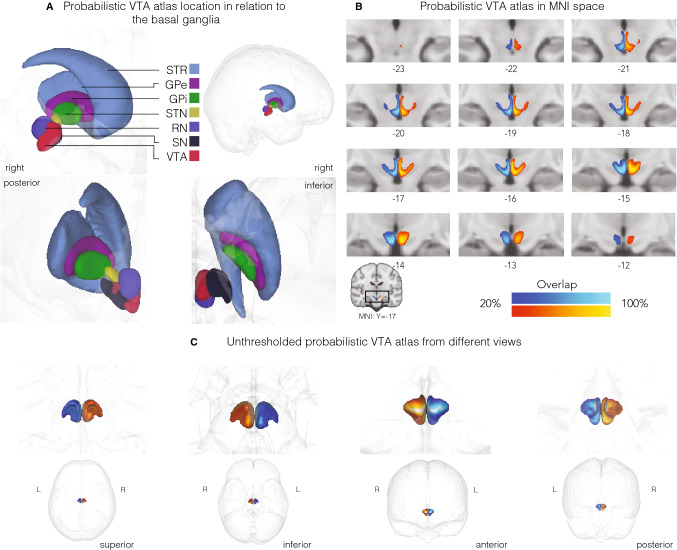
Probabilistic atlas of the VTA. **a** Probabilistic VTA atlas location in relation to the basal ganglia. **b** Thresholded (20%) probabilistic VTA atlas in standard MNI152 2009b space from posterior to anterior direction. **c** Unthresholded probabilistic VTA atlas in glass brain from different views. Left: VTA atlas is depicted in blue and right: VTA atlas in red. Numbers represent slice location with respect to the MNI *Y*-coordinates. The color gradient indicates overlap across the 27 participants with lighter colors indicating a larger overlap (up to 100%) and darker colors less overlap (with a minimum of 20%). *STR* striatum, *GPe* globus pallidus externa, *GPi* globus pallidus interna, *STN* subthalamic nucleus, *RN* red nucleus, *SN* substantia nigra, *VTA* ventral tegmental area

The unthresholded probabilistic atlas volume was 2226.375 mm^3^ for the left and 2368.00 mm^3^ for the right hemisphere. To increase the probability of the atlas voxels belonging to the VTA, thresholds can be applied. Each voxel has a particular probability of belonging a structure, based on the overlap across the 27 individually segmented masks. A threshold of 50%, for example, excludes all VTA atlas voxels that are not shared across at least 50% of the subjects (see Fig. 3 for the volume of the probabilistic atlas across different lower thresholds and Fig. 4 for three-dimensional reconstructions of the atlas).

**Fig. 5:**
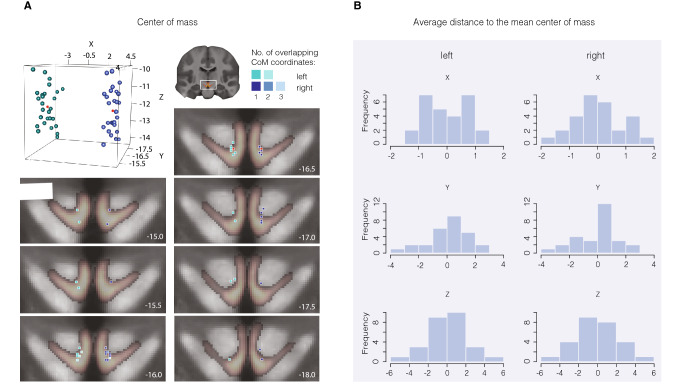
Location of individual VTA masks in standard space. Panel **a** shows the center of mass (CoM) location of each individual conjunction mask in MNI152 2009b space in a three-dimensional graph and the corresponding coordinate overlaid on the probabilistic atlas with a threshold of 20% overlap. CoM coordinates of the left masks are illustrated in green. Right coordinates in blue, and group means in red. As some coordinates fall in the exact same location, the numbers 1, 2 and 3 in **a** [‘Overlap in center of mass coordinates’] represent the colour-coded overlap of individual CoM coordinates, of the adjacent MRI images*.* Numbers in the bottom right corner of the anatomical scans represent the anatomical scanner location of the *y*-coordinate and thus, the images depict midbrain slices from anterior to posterior direction. Panel **b** illustrates the average distance to the overall mean CoM for each coordinate and hemisphere, respectively

Mean center of mass (CoM) of the individual masks in MNI152 2009b space was *x* = − 3.44, *y* = − 16.47, *z* = − 12.03 mm, respectively, for the left VTA masks and *x* = 3.5, *y* = − 16.45, *z* = − 11.99 mm, respectively, for the right VTA masks. The average distance to the mean CoM of the left VTA masks was < 0.001 mm (SD = 0.77) for the CoM *x*-coordinate, < 0.001 mm (SD = 1.42) for the CoM *y*-coordinate and < 0.001 mm (SD = 2.14) for the CoM *z*-coordinate (Fig. 5). Right VTA masks elicit an average distance to the mean *x*-coordinate of < 0.001 mm (SD = 0.86), < 0.001 mm (SD = 1.42) to the mean *y*-coordinate and < 0.001 mm (SD = 2.23) to the mean *z*-coordinate (see Fig. 5, panel b). Analyses in MNI152 2009b space revealed no effects of gender or hemisphere on mask volume (gender: *F*(1, 50) = 0.298, *p* = 0.588, hemisphere: *F*(1, 50) = 0.076, *p* = 0.784; gender × hemisphere: *F*(1, 50) = 0.203, *p* = 0.654).

The mean Dice coefficient was 0.56 (SD = 0.06) for the left VTA and 0.56 (SD = 0.07) for the right VTA, while the Hausdorff distance was 7.96 voxels (SD = 1.34) [5.57 mm, SD = 0.94] and 8.12 voxels (SD = 1.48) [5.68 mm, SD = 1.04], respectively. The dilated Dice coefficient was 0.80 (SD = 0.07) for both hemispheres (see Fig. 4 for the probabilistic atlas).

**Fig. 6:**
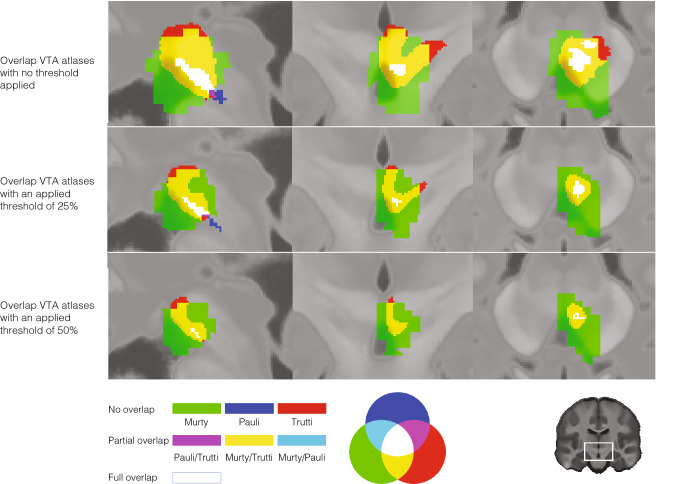
Overlap of proposed new probabilistic VTA atlas with existing probabilistic VTA atlases. The first row shows the overlap between the new probabilistic atlas of the VTA (red), the VTA atlas by Murty et al. (2014; green) and Pauli’s et al. (2018) VTA atlas (blue). The other colours indicate the overlap according to RGB-contrast as depicted in the legend at the bottom of the figure. For reasons of clarity, only the right VTA atlas is shown (at MNI *Y*-coordinate: − 17.0). The second and third rows are identical to row one but illustrate atlases with a threshold of 0.25 and 0.50, which excludes voxels with a probability of belonging to each of the three VTA atlas masks of less than 25% or 50%, respectively, to assess general overlap as well as finer differences. For all shown images MNI *y*-coordinate was at − 17

Table 1 shows the results of the comparison of the proposed atlas with currently available probabilistic atlases of the VTA in MNI152 2009b space (see Fig. 6 for illustration).

**Table 1:** Differences of novel probabilistic VTA atlas to existing probabilistic atlases of VTA

	Reference atlas	Side	Volume difference^a^	Dice overlap	Dilated dice overlap
Threshold at 0%	Pauli et al. (2018)	Left	0.937	0.104	0.170
		Right	0.894	0.172	0.249
	Murty et al. (2014)	Left	0.302	0.576	0.678
		Right	0.273	0.592	0.689
Threshold at 25%	Pauli et al. (2018)	Left	0.879	0.143	0.248
		Right	0.866	0.154	0.264
	Murty et al. (2014)	Left	2.089	0.379	0.507
		Right	2.199	0.385	0.513
Threshold at 50%	Pauli et al. (2018)	Left	0.921	0.108	0.195
		Right	0.909	0.110	0.209
	Murty et al. (2014)	Left	3.021	0.312	0.457
		Right	3.142	0.316	0.456

^a^In mm^3^

## Discussion

In the present work, we propose a new probabilistic atlas of the VTA that, differently from previously proposed atlases, not only takes into account individual variability and precision but explicitly emphasizes it. This atlas provides researchers with a tool to investigate with high anatomical precision the cognitive processes underlying VTA activation using fMRI. We show that ultra-high field 7T MRI allows the identification and delineation of the VTA at the individual level, with the help of optimized MR sequences and a multimodal imaging approach.

This atlas differs from existing work in multiple respects. Although this atlas is based on a fewer number of subjects compared to Murty et al. (2014) and Pauli et al. (2018), it takes anatomical variability more comprehensively into account. First, the tailored MRI contrast elicits more CNR compared to a standard T1-weighted scan and an entirely landmark-based approach as applied by Murty et al. (2014), rendering it more likely to capture true individual anatomical differences even though Murty et al. (2014) had almost double the sample size. Second, Pauli et al. (2018) performed delineations not on individual subjects, but on eight groupwise averages of 168 subjects. Such large groupwise averages capture very little anatomical variability and are biased towards more consistent labeling.

In this study, we used a high-resolution multi-parametric quantitative MRI sequence, which may not be available on every MRI machine. The key features for successfully imaging the VTA were to combine a T1-weighted image (identifying ventricle boundaries) with a T2*-weighted image (identifying SN and RN). Thus, alternative MRI sequences that combine these two contrasts at high isotropic resolution could be used as a basis for VTA delineation, and might be achievable at lower field strength and clinically acceptable scan times. Yet, our MRI sequence enabled segmentation on images with not only increased CNR but also increased resolution and SNR. All these factors play a crucial role in the identification and delineation of subcortical structures, especially structures that lack clear borders and elicit complex shapes, such as the VTA. The influence of the data quality on the VTA mask can be seen in Fig. 6 with respect to the difference of VTA mask volume of Murty et al. (2014; shown in green) and our VTA mask (shown in red). Besides the illustration of the observable volume differences between the atlases, Fig. 6 and Table 1 show various differences across available probabilistic VTA atlases. First, the anatomical precision of the Murty atlas appears constrained as it overlaps largely with areas that do not represent brain tissue but parts of the ventricle system (i.e., fourth ventricle), under all three visualized thresholds. Further, it covers a large region of the midbrain and, as noticeable for the anatomically-experienced reader, includes structures such as the RN or SN. As mentioned above, this affected by data quality with respect to resolution, CNR and SNR that fundamentally affect anatomical precision, something that we could take advantage of compared to Murty et al. (2014) who could not benefit from recent technological advances. Yet, the overlap of Murty’s atlas and the new atlas (shown in yellow in Fig. 6) is substantially more compared to the overlap with the atlas by Pauli et al. (2018; shown in white/blue in Fig. 6). One reason for the difference is the applied VTA terminology, nevertheless, the Pauli atlas is substantially smaller. Hence, second, differences in terminology have strong effects on size, shape and location of the atlases. While we explicitly determined our delineating region according to the ‘VTA region’ terminology, Pauli et al. (2018) evidently delineated a VTA matching a ‘VTA nucleus’ terminology and, with the PBP nucleus, essentially segmented two nuclei that fall within the ‘VTA region’. Although not discussed in the paper, it appears Murty et al. (2014), in line with Ballard et al. (2011), opted for the more established ‘VTA region’ terminology as well (Trutti et al. 2019). Since the area the atlas covers matches previous studies that applied the same terminology (Ballard et al. 2011; Eapen et al. 2011; Murty et al. 2014), and the individual mask volume (~ 138 mm^3^) practically equals the post-mortem reconstructions of VTA volume (Halliday and Törk 1986), in contrast to previously published probabilistic VTA atlases (Table 1, Fig. 6), this atlas meets the goal of anatomical precision. A third major difference across the atlases, observable from Fig. 6 and Table 1, is the size of the atlas after thresholding. In practice, atlases are commonly used with an applied threshold of > 30–50% for neuroimaging analyses. The Pauli atlas is extremely small with a threshold of 50%; thus, it is questionable if it represents a utilizable ROI mask in practice.

One shortcoming of the proposed atlas is noticeably that two of the inter-rater estimates indicate rather low reliability of the delineation. There are multiple sources for the bias in volume difference between the two raters. First, technical limitations and also anatomical constraints make it difficult to precisely capture the anatomy of the VTA, just as for any subcortical structure. As discussed earlier, as well as in Trutti et al. (2019), the borders of the VTA are very hard to identify and the complex shape of the VTA further complicates the identification of the VTA’s borders. All these factors make delineation very challenging, as identification of the border is a crucial basis for overlap, which is reflected in the relatively low Dice scores. Second, the large surface of the VTA due to its complex shape leaves much room for disagreement among raters, an effect that we tried to quantify with the dilated Dice coefficient and visualized in Fig. 2. The dilated Dice score suggests much more overlap (24% more overlap compared to the classical Dice score). Additionally, the Dice score is influenced by the volume of the region as it is computed based on the ratio between the overlap and junction of the masks, which generally leads to lower scores in smaller regions. Third, a difference in expertise in subcortical anatomy and segmentation of subcortical nuclei was present across the raters and likely reflects another source of variance. For example, one rater was well experienced with the segmentation of subcortical nuclei and has been involved in work on the subthalamic nucleus before. As the subthalamic nucleus represents a dorsolateral landmark that is difficult to identify for the unexperienced rater, the ability to correctly identify it as a landmark could be one explanation for the volume difference between raters. In fact, the main difference in segmented voxels between raters is located in the dorsolateral extend of the VTA.

Again, when taking into account a single voxel of uncertainty with the dilated Dice coefficient, only minor differences in the overlap between the raters are indicated (mean overlap of 80%, Fig. 2). This suggests that differences between raters are mostly within one voxel, reflecting the difficulty to precisely locate the boundary, but that the overall location, shape, and size of the VTA is consistently estimated by both raters. Thus, for complex-shaped and difficult identifiable structures such as the VTA, the classical Dice coefficient might only poorly reflect the spatial overlap between the raters, and, consequently, the dilated Dice coefficient is likely to be the better metric for these structures.

Another point worth discussing is the distribution of probability labels in the VTA atlas, with lower probability voxels in the dorsomedial and -lateral VTA. Since all intra-subject measures were performed in subject space, the probability distribution as such cannot be introduced or influenced by registration. Hence, the fact that the low probability voxels are found in the distal ends of the VTA likely reflects the increased difficulty in manually segmenting the dorsomedial and -lateral extensions of the VTA which, as a consequence, affects agreement and spatial overlap. One influencing factor is that the VTA elicits less noisy signal in the ventromedial VTA compared to the dorsomedial and -lateral VTA which, in turn, facilitates manual segmentation of that area. More precisely, the ventro-medial landmarks are defined based on CSF contrast in the fourth ventricle which is sharp and precise on T1-weighted MRI. T2* imaging, which served as a basis for identification of the landmarks SN and RN, is generally less precise due to non-local and orientation-dependent effects, and the SNR is typically lower in such iron-rich regions. Thus, although the ventro-medial VTA has a much more diverse neural composition, its defining landmarks are more visible. In addition, the PBP nucleus which makes the largest part of the dorsolateral VTA has a patchy appearance similar to the SN, which makes defining its boundaries more challenging. Another factor likely relating to the spatial differences in probability is that the medioventral VTA is the VTA region with the largest volume on MR images as it covers a region of multiple component nuclei, such as the VTA nucleus, the PN, the IF, ventral parts of the linear raphe as well as the ventromedial tip of the PBP nucleus (Halliday and Törk 1986; Trutti et al. 2019). Agreement in spatial overlap is critically affected by a structure’s volume and the extent to which this volume affects agreement scores such as Dice is depicted in Fig. 2. Additionally, the ventromedial VTA has a less complex, ‘arborized’ (more ‘spherical’) shape which hugely facilitates segmentation and consequently spatial overlap.

Taken together, these factors make it more likely that the individual masks overlap in the ventromedial VTA region. Consequently, they overlap less in the extensions of the VTA in which segmentation is more difficult due to noise in the signal and volume of the region-of-interest in general.

In summary, this probabilistic atlas of the VTA contributes to a large body of work in atlasing the subcortex by means of manual segmentation on ultra-high-field 7T MRI data. To date, many in vivo studies on the functions of the dopaminergic midbrain nuclei neglect the anatomo-functional differentiation of the VTA and SNc (e.g., Hauser et al. 2017; D’Ardenne et al. 2012) which might lead to misleading outcomes. Although there is likely to be functional and structural overlap between VTA and SNc, their differentiation has been well established, especially in humans (Fu et al. 2016; Murty et al. 2014; Dombug and ten Donkelaar 1991; Halliday and Törk 1986). Yet, we acknowledge that the lack of common nomenclature in combination with data quality that does not allow distinction in the ventral tegmentum (Trutti et al. 2019; Fu et al. 2016; van Domburg and ten Donkelaar 1991; Morales and Margolis 2017) might have hampered the anatomo-functional differentiation reported in studies considered here. This lack of agreement in terminology and consequently, in the applied atlas, has been our main motivation to construct a probabilistic atlas of the VTA, as anatomically precise reconstructed ROI has the potential to overcome anatomical misconceptions, while at the same time making a ROI available that allows comparison of research findings (provided the use of the same atlas). The fact that the average individual mask volume matches post-mortem estimations of VTA volume in a study that applied the same terminology (Halliday and Törk 1986) represents notable support for the anatomical precision of our individual segmentations and consequently, probabilistic atlas.

### Limitations

The atlas can be useful for most MRI studies within the cognitive neurosciences because it is based on healthy young participants and therefore covers a widely studied age group. However, given the well-known age-related changes in subcortical gray matter (Han et al. 2018; Keuken et al. 2017; Good et al. 2001), users should be cautious with applying this atlas to other age groups.

Further, a common trade-off in 7T imaging is that the benefit of an increased SNR in 7T often comes at the cost of substantially increased artifacts due to long scanning times. The MP2RAGE-ME sequence (Caan et al. 2018) chosen for this study, is a very time-efficient technique, acquiring the images of interest (T1-weighted and T2*-weighted) simultaneously, slice-by-slice. In a recent study (Bazin et al. 2016), the amount of blurring due to head motion was very limited around the fourth ventricle, indicating a better performance of the sequence compared to more dorsal regions.

For the research field, common constraints are the limited availability of 7T MRI scanners, the more challenging development of sequences and processing of data make it difficult for researchers to work with 7T data, resulting in many publications that are based on 3T data. In fact, the subcortical nuclei such as the VTA are visible also in (optimised) 3T data, but the anatomical precision remains largely inferior to optimised 7T protocols (Isaacs et al. 2020). Precisely, due to a weaker field strength, 3T (or lower) might not succeed to image with enough spatial resolution, signal and contrast to capture the local neuroanatomy sufficiently well. Accordingly, 3T data certainly allows imaging of the midbrain structures but especially in the case of the heterogenous neurons that make up the VTA, the anatomical precision is considerably impaired. Note that, even with 7T the nature of the VTA makes it difficult to reconstruct it easily and reliably. Given our access to state-of-the-art data and previous efforts and experience with subcortical imaging and segmentations, we were motivated to provide the research field an anatomically precise atlas of the VTA that can also be applied to other, i.e., 3T, data. This way, the new atlas can provide anatomical precision that is hard to achieve with lower field-strength MRI.

### Future perspectives

Our atlas offers the opportunity for several exciting new research directions ranging from preparation to analysis stages of (f)MRI studies. For example, at the piloting stage, it can be applied as a ROI to compare temporal signal-to-noise ratio (tSNR) of different functional MRI protocols (de Hollander et al. 2017) or SNR and CNR in structural MRI protocols. Further, this probabilistic VTA atlas allows investigating functions associated with the dopaminergic midbrain, such as the debate of clear functional distinction between the VTA and SNc in humans (Trutti et al. 2019). For instance, similar to Murty et al. (2014), future studies could compare functional resting-state networks associated with the VTA and SNc, respectively (for more details on such an approach, see de Hollander et al. 2017), or localize specific signals such as the dopaminergic reward prediction error using reward-based tasks in the scanner (Fontanesi et al. 2019).

Cytoarchitectural differences in the dopaminergic system as seen in rodents and behavioral, motor and/or cognitive correlates of such that are also found in humans, are suggested to be driven by, e.g., gender (for a comprehensive review see Gillies et al. 2004, 2014a,b; Gillies and McArthur 2010a, b) and handedness (Barnéoud et al. 1990; Cabib et al. 1995; Sun and Walsh 2006). Application of the tailored MRI midbrain contrast and segmentation protocol suggested by this study makes it possible to study the effects of gender and handedness on the anatomy of the dopaminergic system and its relationship with human behavior. Yet, such studies will require larger samples than this study was able to provide and equal group sizes to test these effects with statistical accuracy.

Further, the application of the segmentation and imaging protocol to middle-aged and elderly subjects represents interesting future research as it will allow testing for age-related effects on, i.e., VTA volume, besides providing data for constructing additional age-specific VTA atlases.

## Conclusion

Although the VTA is a small midbrain structure with complex neurochemistry, anatomical features and shape, using high-field 7T MRI together with optimized MR sequences and a multimodal approach it is possible to better identify and delineate the VTA. Accordingly, we developed a probabilistic VTA atlas specifically for the most studied age group in psychological studies: young-aged subjects between 18 and 30 years of age. The relatively low inter-rater agreement (see Results) highlights the challenges and limitations associated with atlasing the VTA, as discussed in a previous publication (Trutti et al. 2019). However, another more flexible spatial-overlap metric, the dilated Dice coefficient, suggests much better overlap and thus, indicates rather successful VTA segmentation. Additionally, further efforts in scan sequence optimization such as SNR and CNR in the subcortex might improve VTA visualization and consequently (manual) delineation in the future.

## Data Availability

The probabilistic VTA atlas is accessible in the Open Science Framework: https://osf.io/9pzj3/. Further requests for the data or materials can be sent via email to the corresponding author.

## Code availability

Code for data processing (computation of the midbrain contrast) and analysis will be made available on github (https://github.com/ACTrutti/VTA-atlas).
